# Laboratory and Field Investigation of the Feasibility of Crumb Rubber Waste Application to Improve the Flexibility of Anti-Rutting Performance of Asphalt Pavement

**DOI:** 10.3390/ma11091738

**Published:** 2018-09-15

**Authors:** Hongyin Li, Hailong Jiang, Wenwu Zhang, Peng Liu, Shanshan Wang, Fei Wang, Jizhe Zhang, Zhanyong Yao

**Affiliations:** 1 Department of Maintenance, Qilu Transportation Development Group, Yinfeng Fortune Plaza D Block, Jinan 250014, China; qypgjj@126.com (H.L.); sddxzjz@sina.com (H.J.); sdjzdxscj@sina.com (W.Z.); sddxlsj@sina.com (P.L.); 15969677683@139.com (S.W.); vdvvfv@163.com (F.W.); 2 School of Qilu Transportation, Shandong University, Jinan 250061, China; zhanyong-y@sdu.edu.cn

**Keywords:** crumb rubber, anti-rutting agent, flexibility, field evaluation

## Abstract

Resistance of asphalt mix to low-temperature cracking and rutting at high temperature is very important to ensure the service performance of asphalt pavement under seasonal changes in temperature and loading. However, it is challenging to balance the improvement of such resistance by using additives, e.g., anti-rutting agent (ARA). This study focuses on improving the flexibility of anti-rutting asphalt mix by incorporating crumb rubber (CR) and ARA. The properties of the prepared modified asphalt mix were evaluated in the laboratory by performing wheel tracking, three-point bending, indirect tensile, and uniaxial compression tests. The experimental results showed that the dynamic stability of modified asphalt mix was significantly increased due to the addition of ARA and further improved by incorporating CR. The maximum bending strain at −10 °C was increased due to the contribution of CR. The results of indirect tensile strength and resilient modulus further indicated that the CR-modified anti-rutting mixture was more flexible. Moreover, the field observation and evaluation indicated that the CR-modified anti-rutting asphalt pavement met the standard requirements, better than normal asphalt mixture in many parameters. A conclusion can be made that incorporating CR in asphalt mixture prepared with ARA can improve pavement performance at both high and low in-service temperatures.

## 1. Introduction

Owing to economic growth and the high demand of transport, especially in developing countries, traffic volume is experiencing a dramatic increase and the number of overloaded vehicles is increasing as well. Consequently, road pavement suffers severe loading conditions, under which severe distress occurs much earlier than its designed service life [1,2]. For instance, the mass of an overloaded lorry in China can be >150 tons, with contact pressure >1 MPa. Rutting, which is defined as permanent deformation along the wheel path caused by traffic loading, is one of the principal distress modes in Chinese asphalt pavement [3]. The presence of rutting not only accelerates already existing distress, but also increases the potential for driving crashes and reduces driving comfort [4].

For asphalt pavement, the hot-mixed asphalt mixture is commonly obtained at high temperatures by mixing predetermined rations of aggregates (coarse and fine), bitumen, and filler, which, after paving and compaction processes, form a flexible pavement [5,6]. As it is a composite material, its aggregate type, aggregate gradation, air void, binder type, and binder content are the primary factors that control rutting resistance [7]. It has been found that aggregate gradation influences the internal structure and stress distribution of asphalt mixture, which in turn affects the intergranular friction of particles inside [8,9]. Furthermore, intergranular friction influences the movement (rotation or repositioning) of aggregate particles, which finally contributes to the rutting resistance of asphalt pavement [10]. During aggregate movement, the slide action of well-bounded particles shear the binder film [3]. For the same aggregate skeleton, asphalt mix produced with a high-modulus bitumen binder results in better rutting resistance [5]. Therefore, enhancing the bitumen properties seems to be a feasible method to provide good resistance to rutting.

Traditionally, the use of polymer-based materials in bitumen incorporated by mechanical mixing or chemical reactions can significantly improve the properties of conventional bitumen binder in asphalt mix [11]. Two types of polymers are widely used for bitumen modification: plastomers and thermoplastic elastomers [12]. Using plastomers and elastomers usually results in improved rutting resistance at elevated temperatures. The common plastomers used for bitumen modification include polyethylene (PE), polypropylene (PP), ethylene-vinyl acetate (EVA), and ethylene-butyl acrylate (EBA) [13]. The addition of plastomers brings high rigidity to the bitumen and substantially reduces its deformation under traffic load [14]. As the dosage of plastomers increases to a certain extent, two interlocked continuous phases are formed and result in substantially improved bitumen modulus [15]. Based on this behavior, plastomers are usually employed as anti-rutting agents, with the view of improving the high-temperature performance of asphalt mix. However, it was found that plastomers can weaken asphalt’s ability to disperse accumulated stress at low temperatures [16]. These plastomers materials thus failed to improve the fatigue and fracture resistance of asphalt mix at low temperatures [17].

With the rapid growth of car ownership, a huge amount of vehicle tire waste is generated. According to some statistics, around 10 billion tires are discarded every year, and their inadequate disposal results in some adverse effects such as fire risk, rodents, soil pollution, and water pollution, which eventually threaten the environment and human health [18]. Crumb rubber (CR), a thermoplastic elastomer that can be produced from end-of-life tires, has been successfully used as an additive for bitumen modification due to its excellent properties and low cost. Previous researchers have reported that CR plays an important role in improving the resistance of permanent deformation of bitumen, fatigue cracking, and crack reflection at both high and low in-service temperatures [19,20]. CR also improved the elastic recovery of bitumen and showed good performance both in the lab and in field tests [21]. However, it seems difficult for CR-modified bitumen to substantially improve the high-temperature performance of asphalt pavement [22]. 

To overcome the drawbacks of common polymer modified techniques and improve the performance of asphalt mixture, an integrated modification method was employed in this study to produce modified asphalt mix by using both anti-rutting agent (ARA) and crumb rubber (CR). The main objective of this paper was to investigate the possibility of using CR to improve the flexibility of anti-rutting asphalt pavement. CR-modified bitumen was prepared by mixing base bitumen with a specific dosage of CR. Then, an asphalt mixture prepared with the modified bitumen was developed and produced with the anti-rutting agent added directly during mixing. The properties of asphalt mix were tested by wheel tracking, three-point bending, indirect tensile strength, and resilient modulus tests in order to evaluate the mechanical performance. Moreover, field tests were carried out on constructed test roads, followed by numerous field measurements, to investigate the properties of the field asphalt pavement.

## 2. Materials and Experimental Methods

### 2.1. Materials

Grade 70 base bitumen with a penetration grade of around 70 dmm produced by Chinese Qilu Transportation Development Group (Jinan, China) was used to produce asphalt mix and test roads. Properties of this bitumen, which were evaluated in accordance with the Chinese JTG E20-2011 standard [23], are presented in Table 1.

The aggregates used in the mix were produced from the Daolang limestone quarry in Taian City, Shandong Province. The physical properties of fine and coarse aggregate were characterized according to the Chinese JTG E20-2011 standard [23], and the results are presented in Table 2. 

The CR used in this research was reclaimed from end-of-life vehicle tires. Tires were first shredded and chipped using large machinery to obtain rubber shreds. Furthermore, the ambient grinding process was implemented to reduce the particle size to 40 mesh, and the physical properties are listed in Table 3. The ARA used in the mixture was supplied by Chinese Shandong Qilufa Transportation Technology Co. Ltd (Dezhou, China). The ARA was derived from reclaimed PE, and its properties are shown in Table 4.

### 2.2. Mix Design

Before preparing the asphalt mix, the CR-modified bitumen was produced by the following procedure. The base bitumen was heated up to 160 °C until it melted. Then, CR in a dosage of 15% by mass was added to the bitumen, followed by a swelling process at 160 °C for 1 h. The CR-modified bitumen experienced a high-speed shearing process at a speed of 5000 r/min for 1 h in order to obtain the essentially homogeneous CR-modified bitumen. 

**Figure 1:**
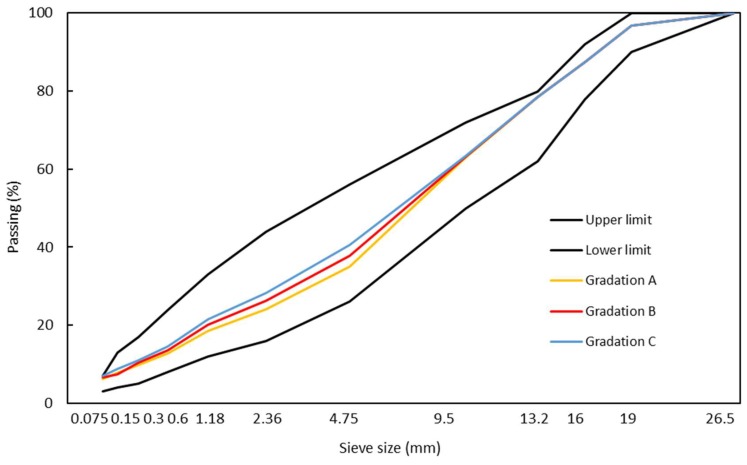
Gradation curves of aggregates used compared with standard requirements.

The asphalt mixture designed in this research was used for the middle layer of asphalt pavement with a maximum particle size of 19 mm. The mixture was designed based on Chinese JTG E20-2011 [23], JTG E42-2005 [24], and JTG F40-2004 [25] standards. Three aggregate gradations were designed, as shown in Figure 1. The ARA content used was 0.3% of the total weight of the mixture and first blended with aggregates before bitumen. This ARA content is the supplier’s recommended optimum dosage. The Marshall specimens were prepared by using 5 different binder contents (3.1%, 3.6%, 4.1%, 4.6% and 5.1%). The best aggregate gradation and optimum binder content were determined by considering targeting air voids of 4.0–4.5% and the highest Marshall stability. Finally, gradation B was selected as the best, and the optimum binder content was found to be 4.3% for base bitumen and 4.4% for CR-modified bitumen. The results of Marshall stability and flow of 3 selected mixes are shown in Table 5.

### 2.3. Laboratory Testing Methods

#### 2.3.1. Wheel Tracking Test

The wheel tracking test (WTT), in accordance with the JTG E20-2011 standard [23], was used to evaluate the rutting resistance of asphalt mix at high loading and high temperature. Owing to its ability to induce a stress state in asphalt slabs similar to that in actual pavement, the WTT was considered to be a suitable method for rutting investigation [26]. Asphalt specimens with dimensions of 300 × 300 × 50 mm^3^ were compacted by a roller compactor (Shandong Luda Test Instruments Co. LTD, Taian, China) to obtain slabs with air voids in the range of 4.0–4.5%. Before testing, these slab specimens were conditioned in an environmental chamber at 60 °C for 6 h. During testing, the contact pressure between rubber wheel and specimen surface was 0.7 MPa, with a loading speed of 42 passes per minute. Two replicated tests were performed on each asphalt mixture. The development of rutting depth was measured and recorded with a linear variable differential transformer (Shandong Luda Test Instruments Co. LTD, Taian, China). The dynamic stability value was calculated by Equation (1):

(1) $$DS=\frac{15N}{d_{2}-d_{1}}\;$$

where *DS* is the dynamic stability (cycles/mm), *N* is the loading speed (42 cycles/min), *d*_1_ is the rutting depth (mm) at 45 min, and *d*_2_ is the rutting depth (mm) at 60 min.

#### 2.3.2. Three-Point Bending Test

**Figure 2:**
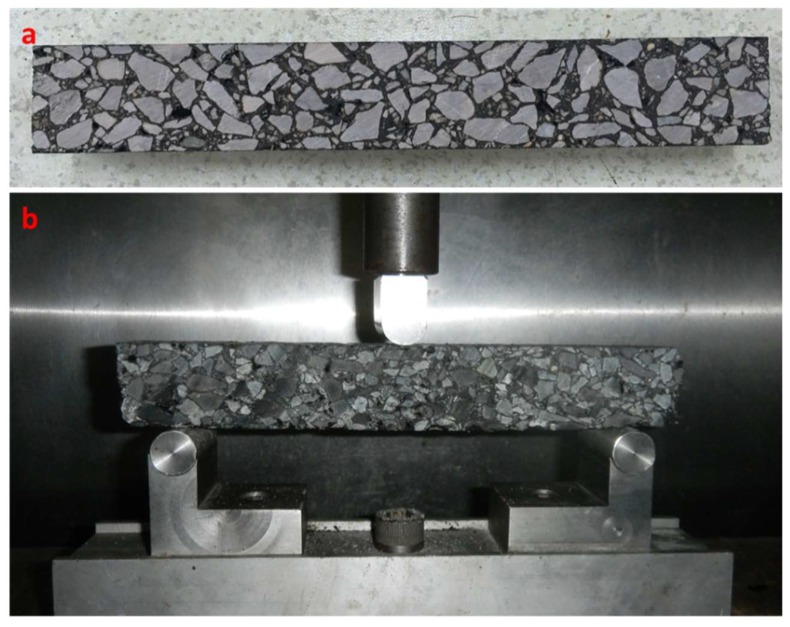
Specimen and setup used for three-point bending test: (**a**) prismatic beam; (**b**) test setup.

The three-point bending (3PB) test is a uniaxial loading system used to evaluate the low-temperature cracking resistance of asphalt mix [27]. The 3PB was performed according to the JTG T0715-2011 standard [23]. Before testing, asphalt mixture slabs were compacted, followed by a cutting process to obtain prismatic beams with dimensions of 250 × 30 × 35 mm^3^, as shown in Figure 2a. The air voids of asphalt mixture beams were measured, and these 3 types of mixture obtained similar average air voids, with values in the range of 4.1–4.5%. Before testing, these mixture beams were placed in an environmental chamber at −10 °C for at least 1.5 h to ensure homogeneous temperature distribution. During testing, the specimen was supported by 2 rollers with a span length of 200 mm, and a concentrated loading rate of 50 mm/min was applied, as shown in Figure 2b. The maximum bending strain was calculated by Equation (2):

(2) $$\varepsilon_{B}=\frac{6\times h\times d}{L^{2}}\;$$

where *ε_B_* is the maximum bending strain (µε), *h* is the height of the specimen (mm), *d* is the deflection of specimens in mid-cross-section (mm), and *L* is the span of the support roller (mm).

#### 2.3.3. Indirect Tensile Strength Test 

The indirect tensile strength (*ITS*) test is commonly used to test the rutting and cracking properties of asphalt pavement [28]. This test was carried out at a loading rate of 50 mm/min and 25 °C with testing procedures according to the JTG T0716-2011 standard [23]. Cylindrical specimens with a thickness of 63.5 ± 1.3 mm and diameter of 101.6 mm were prepared using the Marshall compacter (Shandong luda test instruments co. LTD, Taian, China). During testing, the vertical compressive load was converted into uniform horizontal tensile stress [1,7]. *ITS* was calculated by Equation (3):

(3) $$ITS=\frac{2P_{\max}}{\pi td}\;$$

where *ITS* is the indirect tensile strength (MPa), *P*_max_ is the maximum applied load (N), *t* is the specimen thickness (mm), and *d* is the specimen diameter (mm).

The moisture resistance of asphalt mixture was also characterized by using the *ITS* test in accordance with the JTG T0729-2000 standard. The moisture-saturated Marshall specimens were conditioned in a refrigerator at −18 °C for 16 h, followed by water soaking at 60 °C for 24 h. Before testing, these specimens were immersed in water at 25 °C for 2 h. Moisture sensitivity of different mixes can be compared using the tensile strength ratio (TSR) value as follows:

(4) $$TSR=TS_{1}/TS_{0}\;$$

where *TS*_0_ and *TS*_1_ are the indirect tensile strength before and after the freeze-thaw cycle, respectively.

#### 2.3.4. Resilient Modulus Test

Resilient modulus (RM) of asphalt mix was evaluated using a uniaxial compression test according to the JTG T0713-2000 standard [23]. It is a commonly used stress-strain measurement to measure the elastic properties of asphalt mix [29]. The compressive strength (P) of asphalt specimen was first measured at 15 °C with a loading rate of 2 mm/min before the RM test. Then, uniaxial loading with 7 steps (0.1 P, 0.2 P, 0.3 P,…, 0.7 P) was applied to the specimen and the resilient deformation of each loading stage (Δ*L*_i_) was recorded after 30 s of unloading. The compressive strength of asphalt mix was calculated by:

(5) $$R_{c}=\frac{4P}{\pi d^{2}}\;$$

where *R*_c_ is the compressive strength (MPa), *P* is the peak loading (N), and *d* is the specimen diameter (mm).

The resilient deformation at loading of 0.5 P was selected to calculate the RM value according to Equation (6):

(6) $$E=\frac{q_{5}\times h}{\Delta L_{5}}\;$$

where E is the compressive resilient modulus (MPa), *q*_5_ is the compressive strength at 0.5 P loading (MPa), *h* is the height of the specimen (mm), and Δ*L*_5_ is the resilient deformation (mm).

## 3. Field Test Road and Evaluation

**Figure 3:**
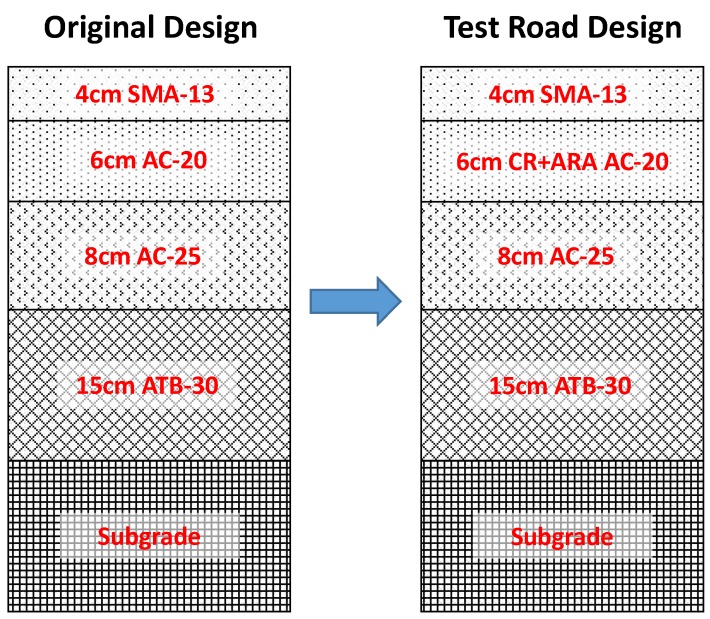
Cross-sections of the road structure: the original designed AC-20 in the middle layer was replaced by CR-modified anti-rutting AC-20.

Based on the CR-modified anti-rutting asphalt mixture designed in the laboratory, a test road was constructed in September 2017. This test road was based on a maintenance project on the S31 expressway with a length of 2.2 km (K6+400~K8+600) in Taian, Shandong Province, China. The maintenance project was designed to remove the top and middle layers of the asphalt pavement, followed by paving with new asphalt mix. The CR-modified anti-rutting asphalt mixture was used in the middle layer with a thickness of 60 mm, as shown in Figure 3. The original designed AC-20 intermediate layer was used as a benchmark to compare with the anti-rutting middle layer.

### 3.1. Production of Asphalt Mix in the Plant

The CR-modified bitumen was prepared by using a reaction kettle (Kaifeng Road construction equipment co. LTD, Dezhou, China) with a capacity of 7 tons, then it was transferred to the bitumen tank and ready for the mixture production. An Ammann-4000 asphalt mixing plant (Ammann Group, Germany), which can produce 3.5 tons of mixture at a time, was used for this project. During the asphalt mixing, the temperature of aggregates and bitumen was controlled in the range of 180–200 °C and 165–175 °C, respectively, to make sure the temperature of the asphalt mixture was not less than 175 °C. The asphalt mixture was dropped onto the lorry immediately after mixing and transferred to the test road for paving. To evaluate the quality of the asphalt mixture, about 30 kg was taken from the lorry for the Marshall stability test and wheel tracking test.

### 3.2. On-site Paving and Compaction

**Figure 4:**
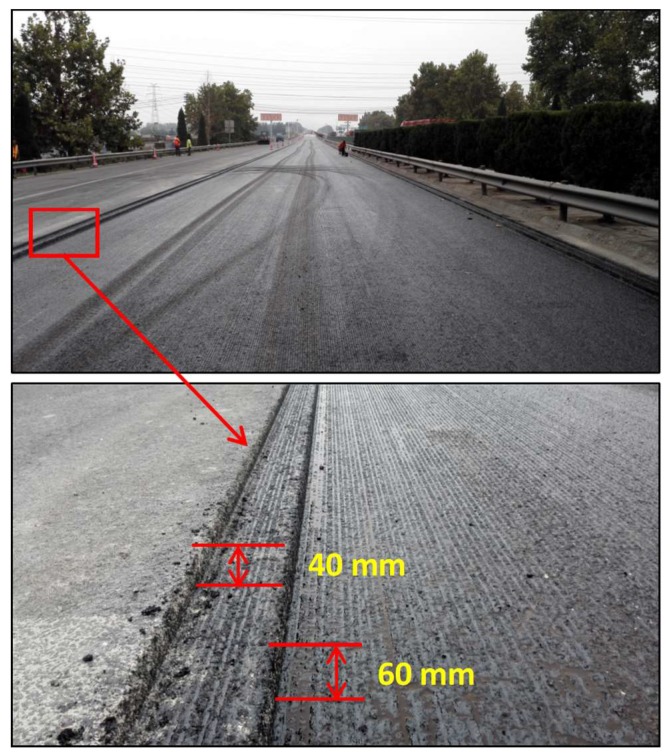
Treatment of underlayer surface with emulsified bitumen to ensure good bonding with the middle layer.

**Figure 5:**
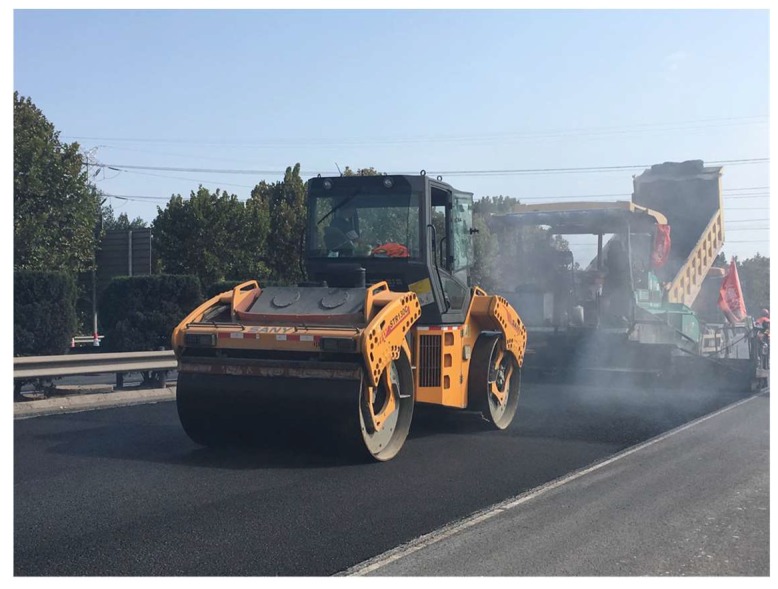
Mechanical equipment used for asphalt paving and compaction: pitch paver and road roller.

Before paving the middle layer, the substrate layer surface was cleaned to remove dust and covered with a layer of emulsified bitumen (Chinese Qilu Transportation Development Group, Jinan, China) to obtain good interface adhesion, as shown in Figure 4. The construction range included a travel lane and an overtaking lane with a width of 7.5 m. Hot mix was first dropped into the pitch paving, with the temperature not less than 165 °C. During the paving process, the coefficient of compaction was 1.34 and the speed was controlled at 3 m/min. Two Sany STR130C-6 (Sany Heavy Industry, Changsha, China) road rollers followed closely behind the pitch paver to finish the first compaction immediately after paving, as shown in Figure 5. At least four cycles of compaction were applied to obtain the required degree of compaction. 

### 3.3. Core Samples from Test Roads

**Figure 6:**
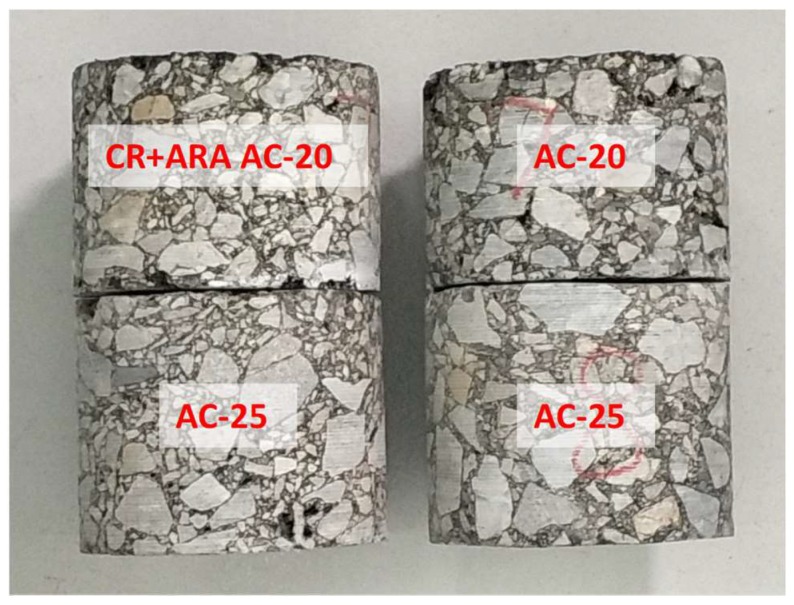
Core samples from the field pavement: (**left**) CR-modified anti-rutting mixture, and (**right**) normal AC-20 mixture.

Eight cored samples with a diameter of 100 mm were collected in four locations after the middle layer construction was completed. Four cores were used to determine the *ITS*, and another four cores were used to evaluate the compressive strength and resilient modulus. Another eight cored samples were collected from the original designed AC-20 layer and the same tests were performed as with the anti-rutting mixture. The cored samples were then cut and trimmed to obtain cylindrical specimens, as shown in Figure 6. The anti-rutting mixture and the normal AC-20 layer were placed on top of the existing AC-25 underlayer. Because of the matching aggregate gradation, the anti-rutting mixture and the normal AC-20 mixture showed almost equivalent skeletal structure.

## 4. Results and Discussion

### 4.1. Laboratory Results

#### 4.1.1. Rutting Resistance and Dynamic Stability of Asphalt Mix

**Figure 7:**
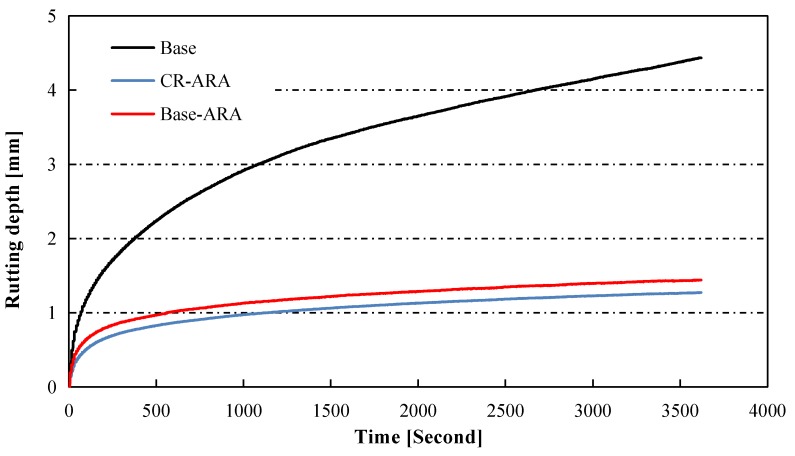
Rutting depth development in asphalt mixture slabs under repeated wheel tracking.

Rutting curves of the three designed asphalt mixes are presented in Figure 7. These three mixes obtained similar air voids.

It can be seen that the rutting depth of asphalt mix increased gradually when the repeated loading time was prolonged, and the base asphalt mixture obtained the greatest rutting depth. The addition of ARA significantly decreased the rutting depth of the asphalt mixture, while the incorporation of CR helped to slightly reduce the rutting depth. 

Dynamic stability (DS), one of the most widely used indicators for evaluating the rutting behavior of asphalt pavement, was calculated based on Equation (1), and the results and standard deviations are presented in Table 6. The dynamic stability of the asphalt mixture prepared with the base bitumen was 1526 cycles/mm, which was the lowest value among the tested specimens. It was found that the dynamic stability of the ARA-modified asphalt mixture was about six times higher than that of the base asphalt mixture, with the value reaching 9324 cycles/mm. However, adding CR did not further enhance the dynamic stability of the ARA-modified asphalt mixture. This indicates that ARA is the main factor in improving the rutting resistance of asphalt mixture. The increased dynamic stability implies that the integrated modification of asphalt mixture using ARA and CR obviously improved the rutting resistance, which in turn contributed to enhanced pavement performance at high temperature.

#### 4.1.2. Maximum Bending Strain of Asphalt Mix

**Figure 8:**
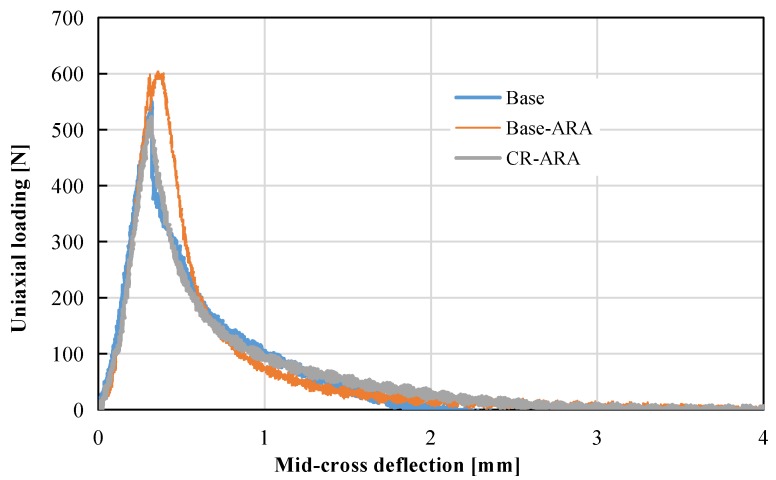
Plots of mid-cross deflection versus uniaxial loading of all specimens.

The stress-strain curves of asphalt mix tested by the three-point bending test are plotted in Figure 8. Maximum bending strain is an indicator of pavement flexibility, and a higher value indicates better resistance to thermal cracking. The results of maximum bending strain are shown in Table 7. In comparison with the base asphalt mixture, the addition of ARA decreased the bending strain from 2067 μm to 1831 μm. However, when CR was added to the mixture, the bending strain showed an obvious increase to 2439 μm. This shows that ARA reduced the deformation capacity of the asphalt mix, which indicates potential resistance to thermal cracking at low temperatures. In contrast, CR improved the flexibility of bitumen and resulted in better low-temperature performance. It can be concluded that the integrated modification of adding ARA and CR improved the low-temperature flexibility of the anti-rutting asphalt mixture. 

#### 4.1.3. Indirect Tensile Strength (*ITS*) Test and Moisture Sensitivity

**Figure 9:**
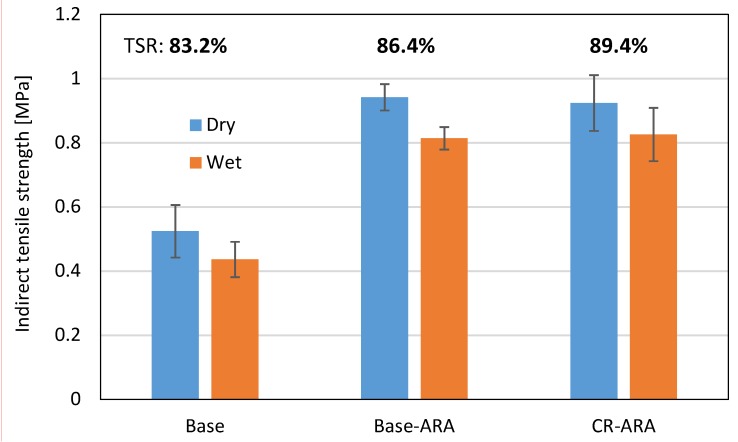
Indirect tensile strength (*ITS*) and tensile strength ratio before and after freeze-thaw cycle.

The indirect tensile strength of asphalt mix before and after the freeze-thaw cycle was measured and the retained tensile strength ratios were calculated, with the results presented in Figure 9. In dry conditions, the asphalt mixture prepared with the base bitumen showed the lowest *ITS* of 0.524 MPa. With the addition of ARA, *ITS* increased to nearly twice that of the base asphalt mixture. This can be attributed to the formation of two interlocked continuous phases in the bitumen, resulting in improved strength of the asphalt mixture. ARA significantly improved the tensile strength of the asphalt mixture, which may contribute to rutting and cracking resistance of asphalt pavement. However, CR-ARA obtained nearly the same *ITS* value as Base-ARA. It is suggested that CR has no further contribution to the *ITS* value of anti-rutting asphalt mixture.

After the freeze-thaw cycle, the *ITS* of all specimens had an obvious reduction due to moisture damage, as shown in Figure 9. Based on the JTG D50-2017 standard [30], the minimum requirement of tensile strength ratio (TSR) for asphalt mix is 75%. As shown in Figure 9, the TSR of the anti-rutting asphalt mixture was greater than that of the asphalt mix prepared with base bitumen, which indicates better resistance to moisture damage. In addition, the CR-modified bitumen resulted in further improvement in TSR. Therefore, the addition of both CR and ARA enhanced the adhesion of aggregate-bitumen interface, which in turn improved the durability of asphalt mixture against moisture.

#### 4.1.4. Compressive Strength and Resilient Modulus of Asphalt Mix

**Figure 10:**
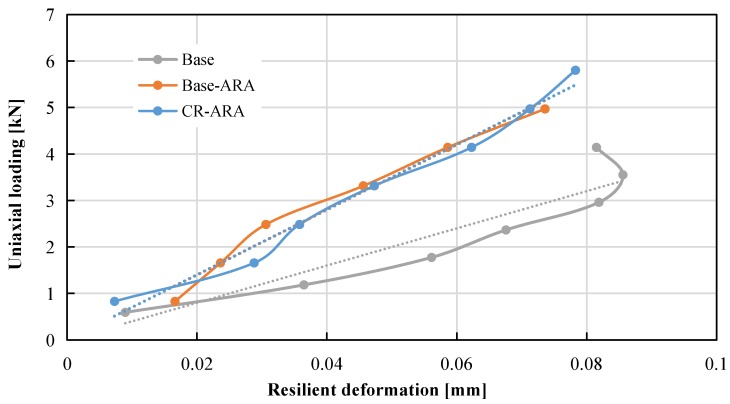
Relationship between uniaxial loading and resilient modulus of all specimens.

**Figure 11:**
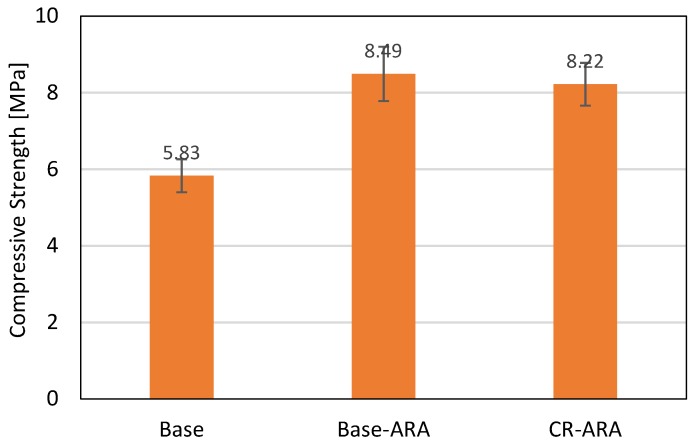
Compressive strength of asphalt mix at 15 °C.

**Figure 12:**
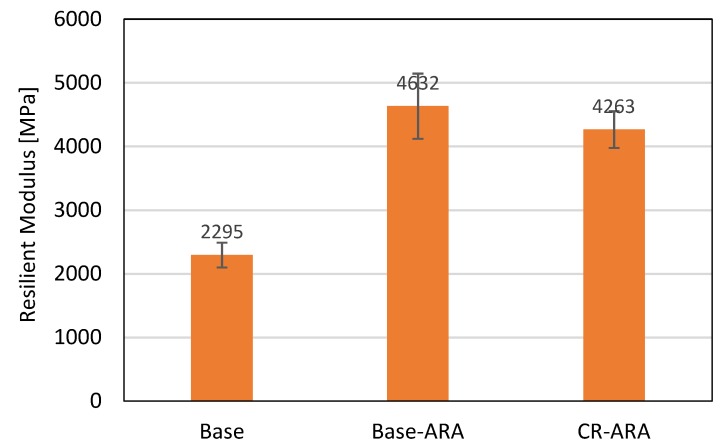
Resilient modulus of asphalt mix at 15 °C.

The relationship between uniaxial loading and resilient modulus of related specimens is shown in Figure 10. It can be observed that the seven points selected in this test are all located on one trend line. The compressive strength and resilient modulus were calculated based on Equations (5) and (6), as shown in Figure 11 and Figure 12, respectively. In terms of compressive strength, the asphalt mixture prepared with the base bitumen obtained the lowest result (5.83 MPa), and the mixture prepared with ARA had the highest (8.49 MPa). Such results indicate that the addition of ARA improved the resistance of asphalt mixture to vertical loading. The addition of CR in the anti-rutting asphalt mixture slightly decreased the compressive strength. Considering the standard deviation, Base-ARA and CR-ARA obtained similar compressive strength. This phenomenon reveals that ARA is the main factor in improving compressive strength and CR does not contribute to this parameter.

As shown in Figure 12, the anti-rutting asphalt mixture obtained the highest resilient modulus value, which means ARA increases the stiffness of asphalt mixture and results in less deformation under loading. However, using CR in the anti-rutting mixture can reduce the resilient modulus, and this can be attributed to the increased recoverable deformation. This demonstrates that adding CR can improve the elasticity of the anti-rutting asphalt mixture. 

### 4.2. Results of Test Road Evaluation

#### 4.2.1. Properties of Asphalt Mix Used in the Test Road

The asphalt mix taken from the mixing plant was tested, and the evaluated properties included Marshall stability and rutting resistance by the wheel tracking test. The properties of the asphalt mix used in the test road are listed in Table 8. As presented in this table, the Marshall stability value of CR-modified anti-rutting mixture was over 30% higher compared with the base asphalt mixture. The flow of the CR-modified anti-rutting mixture was slightly lower than that of the base asphalt mixture. This phenomenon indicates that the integrated modification led to the increased strength of the asphalt mixture and contributed to the high-temperature performance of asphalt pavement. In terms of the wheel tracking results, the dynamic stability of the CR-modified anti-rutting mixture was about six times higher than that of the base asphalt mixture. This result shows the enhancement of the rutting resistance of the CR-ARA mixture. The results obtained from the plant-mixed asphalt mixture had good correlation with the experimental results, as shown in Table 5 and Table 6. This indicates that the integrated modification of using ARA and CR improved the pavement performance in the test road.

#### 4.2.2. Properties of Cored Specimens from Test Road

**Figure 13:**
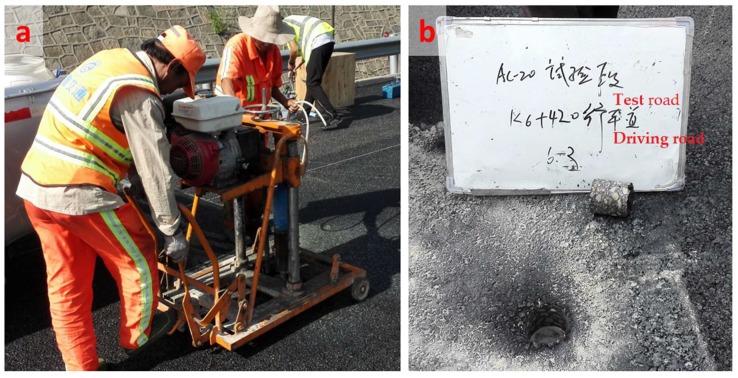
Equipment used for on-site coring and measurement: (**a**) core-drilling machine; (**b**) information of cored specimen.

After test road compaction, the cored specimens were collected by using a core-drilling machine, as shown in Figure 13a. The core locations and thicknesses of cored specimens were recorded, as shown in Figure 13b. The properties of the cored specimens were evaluated, and the results are presented in Table 9. The degree of compaction was the proportion of bulk specific gravity of the cored specimen divided by bulk specific gravity of the Marshall specimen. As presented in this table, the degree of compaction and thickness of these two test roads met the design requirements. The *ITS* results were similar to the laboratory study, which indicates that the CR-ARA asphalt mixture had a higher value. The addition of ARA improved the stiffness of bitumen, which resulted in enhanced strength of the asphalt mixture. However, in terms of the same asphalt mixture, the cored specimen obtained relatively lower *ITS* value compared with the laboratory specimen. One reason could be the specimen dimensions, with the height and diameter of cored specimens differing from those of the Marshall specimen. Another reason could be that the cored specimen destroyed the continuous structure of the compacted road, and the aggregates on the side walls were not restricted by binders. Regarding the compressive strength and resilient modulus, higher values were also related to the CR-ARA asphalt mixture. The improvement of compressive strength and resilient modulus can be attributed to two major factors. First, the addition of ARA (plastomers) creates two interlocked continuous phases and results in increased stiffness. Second, owing to the contribution of CR, a rubbery support network is formed and results in improved elastic response [12]. As a consequence, it can be anticipated that the CR-modified anti-rutting asphalt pavement would be more durable than the base asphalt pavement.

## 5. Conclusions

Recent efforts to develop anti-rutting asphalt pavement show that the addition of ARA failed to maintain good low-temperature pavement performance. This paper investigated the feasibility of using CR and ARA to improve the rutting resistance and flexibility of asphalt pavement, which were evaluated by laboratory tests and field evaluation. The first phase of this study was to clearly evaluate the mixture properties before and after incorporating CR at both low and high service temperatures. The second phase was to construct test roads and detect the related pavement performance. The major findings can be summarized as follows:

1) The addition of ARA significantly reduced the rutting depth and improved the dynamic stability of asphalt mix. This indicates improvement of asphalt mix under high-temperature conditions. Moreover, the dynamic stability of asphalt mixture can be further improved by incorporating CR.
2) ARA can decrease the maximum bending strain, and incorporating CR can obviously increase the maximum bending strain of asphalt mix prepared with ARA. This implies that adding CR can improve the flexibility of anti-rutting asphalt mixture at low temperature.
3) Using ARA in asphalt mix can obviously improve the *ITS* value, resilient modulus, and moisture resistance. The addition of CR into the anti-rutting asphalt mixture can slightly reduce the *ITS* and resilient modulus.
4) The properties of asphalt mix produced by the asphalt mixing plant for field tests had good correlation with the mix prepared in the laboratory. The quality of asphalt mix used for test roads met the standard requirements.
5) The results of *ITS* and resilient modulus of the specimens cored from test roads showed a similar trend to the results obtained in the laboratory, which indicates that the CR-modified anti-rutting asphalt pavement showed the best pavement performance.

It should be noted that the test roads were just constructed, service performance cannot be evaluated over the short term, and more tests should be carried out in the future. Therefore, long-term tracking observation will be performed to characterize the properties of test roads, which in turn can provide guidelines for further research. 

## Figures and Tables

**Table 1:** Physical and chemical properties of bitumen.

Bitumen Test	Result	Technical Requirements	Test Standard
Softening point (°C)	49.0	≥46	T0606
Penetration (25 °C, 0.1 mm)	68	60–80	T0604
Ductility (15 °C, cm)	>100	≥100	T0605
Viscosity at 60 °C (Pa·s)	198	≥180	T0625
Flash point (°C)	295	≥260	T0611
Solubility in C_2_HCl_3_	99.73	≥99.5	T0607
Relative density at 15 °C	1.004	–	T0603

**Table 2:** Physical properties of coarse and fine aggregates.

Aggregate Test	Results		Technical Requirements	Test Standards
	Coarse Aggregate	Fine Aggregate		
Specific gravity (g/cm^3^)	2.738	2.725	≥2.5	T0304/T0328
Water absorption (%)	0.44	0.73	≤3.0	T0304/T0328
Los Angeles abrasion loss (%)	15.4	–	≤30	T0317
Crushing value (%)	19.3	–	≤28	T0316

**Table 3:** Properties of crumb rubber (CR) used in this research.

CR Test	Result	Technical Requirements
Relative density	1.21	1.10–1.30
Moisture content (%)	0.08	0.5
Metal content (%)	0.005	0.05
Fiber content (%)	0.12	0.5

**Table 4:** Properties of anti-rutting agent (ARA) used in this research.

ARA Test	Result	Technical Requirements	Test Standards
Particle mass (g)	0.023	≤0.03	JT/T860.1
Gravity (g/cm^3^)	0.946	≤1.0	GB/T 1033
Melt index (g/10 min)	1.3	≥1.0	GB/T 3682
Ash content (%)	3.3	≤5	JTG E20

**Table 5:** Results of Marshall stability test for selected mix.

Mixture Type	Stability (kN)	Flow (mm)
Base	10.2	2.44
Base-ARA	16.4	1.81
CR-ARA	15.2	2.19

**Table 6:** Dynamic stability of asphalt mix after wheel tracking test.

Property	Base	Base-ARA	CR-ARA
Dynamic stability (cycles/mm)	1526 ± 119	9324 ± 291	9631 ± 317

**Table 7:** Maximum bending strain (*ε_B_*) of asphalt mix at −10 °C.

Property	Base	Base-ARA	CR-ARA
Maximum bending strain (μm)	2067 ± 77.9	1813 ± 92.6	2439 ± 124.5

**Table 8:** Marshall stability and wheel tracking results of mix obtained from mixing station.

Experimental Test	Result		Technical Requirement
	Base	CR-ARA	
Stability (kN)	10.7 ± 0.43	14.1 ± 0.84	≥8
Flow (mm)	2.87 ± 0.32	2.19 ± 0.19	1.5–4
Dynamic stability (cycles/mm)	1732 ± 112	10216 ± 568	≥1000/≥2800

**Table 9:** Related properties of cored specimens from the test road.

On-Site Test	Results		Technical Requirements
	Base	CR-ARA	
Compaction degree (%)	98.6 ± 0.5	98.4 ± 0.6	≥98
Thickness (mm)	63 ± 0.2	65 ± 0.2	≥60
*ITS* (MPa)	0.457 ± 0.042	0.839 ± 0.034	–
Compressive strength (MPa)	3.15 ± 0.40	4.40 ± 0.51	–
Resilient modulus (MPa)	1209 ± 97	2923 ± 304	–
